# Examining the impact of clinical features and built environment on risk of hospital onset *Clostridioides difficile* infection

**DOI:** 10.1017/ice.2024.239

**Published:** 2025-03

**Authors:** Priti Singh, Endia Reid, Justin Smyer, Jennifer Martin, James Odei, Courtney Hebert, David Kline

**Affiliations:** 1 Department of Biomedical Informatics, The Ohio State University Wexner Medical Center, Columbus, OH, USA; 2 Department of Clinical Epidemiology, The Ohio State University Wexner Medical Center, Columbus, OH, USA; 3 Division of Biostatistics, College of Public Health, The Ohio State University, Columbus, OH, USA; 4 Department of Internal Medicine, Division of Infectious Disease, The Ohio State University Wexner Medical Center, Columbus, OH, USA; 5 Department of Biostatistics and Data Science, Wake Forest University, School of Medicine, Medical Center Blvd, Winston-Salem, NC 27157, USA

## Abstract

**Objective:**

Environmental features of a patient’s room depend on the patient’s level of acuity and their clinical manifestations upon admission and during their hospital stay. In this study, we wish to apply statistical methodology to explore the association between room features and hospital onset infections caused by *Clostridioides difficile* (HO-CDI) while accounting for room assignment.

**Method:**

We conducted a nested case–control study using retrospective electronic health record (EHR) data of patients hospitalized at the Ohio State University Wexner Medical Center (OSUWMC) between January 2019 and April 2021. We collected clinical information and combined that with room-based information, collected as surveys. Data were analyzed to assess the association between room factors and HO-CDI.

**Results:**

2427 patients and 968 unique rooms were included in the study. Results indicated protective effects for rooms with cubical curtains near the patient (OR = 0.705, 95% CI = 0.549–0.906), rooms with separate shower units (OR = 0.674, 95% CI = 0.528–0.860), rooms with wall-mounted toilets (OR = 0.749, 95% CI = 0.592–0.950), rooms with sliding bathroom doors (OR = 0.593, 95% CI = 0.432–0.816), and sliding door knobs (OR = 0.593, 95% CI = 0.431-0.815). Rooms with manual paper towel dispensers had increased odds of HO-CDI (OR = 1.334, 95% CI = 1.053–1.691) compared to those with automatic towel dispensers.

**Conclusion:**

Results suggest possible association between specific room features and HO-CDI, which could be further investigated with techniques like environmental sampling. Moreover, findings from the study offer valuable insights for targeted intervention measures.

## Introduction

Environmental factors, such as room surfaces and features, can contribute to the transmission of hospital-onset *Clostridioides difficile* (*C. diff)* (HO-CDI) infections.^1–3^ Because *C. diff* spores are difficult to kill and can persist in the environment for extended periods, they can contribute to in-hospital transmission.^2,4,5^ Additionally, certain rooms or environmental features are more prone to harbor infectious organisms than others, thus further amplifying the risk.^6^ For example, studies conducted by Ching et al^7^ and Jou et al^8^ suggested that the presence of curtains near the patient bed acts as a barrier for disease transmission, while rooms with larger square footage were associated with a greater risk of HO-CDI respectively.

Studying room feature risk is complex as patients are not randomly assigned to rooms, rather patients with similar clinical conditions are often co-located within hospital units. Rooms of co-located patients are more likely to share similar features, compared to rooms in different units or buildings. Previous studies have focused on either clinical conditions or room features alone when assessing risk of HO-CDI, without considering them in the same model.^9^ Therefore, in this study, we wish to explore the association between room features and risk of HO-CDI, when accounting for room assignment factors (patient and visit factors).

**Figure 1.**
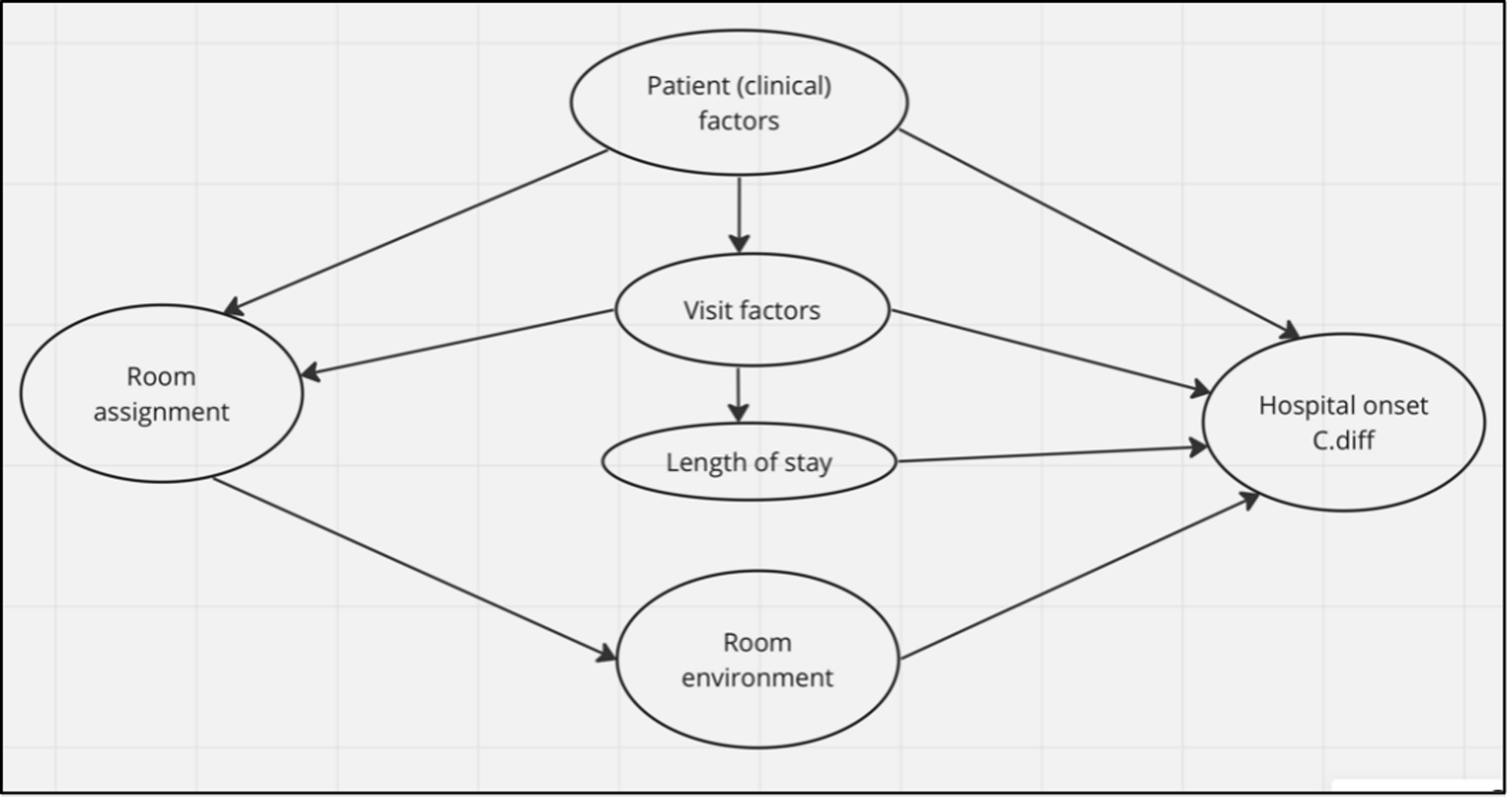
A graphical representation of the key variables and their relationship, as examined in this research.

Figure 1 is a directed acyclic graph (DAG), which helps to visualize the interplay between variables and how they might contribute to the outcome of interest. This DAG guided our selection of co-variates for adjustment in our model.^10^ The decision to assign patients to a particular building, floor, or unit is often made based on their presenting complaint and underlying comorbidities. During their hospital stay, treatment outcomes may necessitate further intra-hospital movement and impact their overall length of stay (the time from admission to discharge). Each of these factors contributes to the risk of HO-CDI and therefore, included in this study.^11–13^ This builds on our research team’s prior exploratory work by employing methods to more rigorously account for room assignment.^2^ In this study we wish to apply statistical methodology to assess the association between room features and HO-CDI while accounting for room assignment.

## Methods

### Data preparation

We conducted a nested case control study. We obtained clinical and patient location information from the electronic health record (EHR). Room environment information was obtained by the research team via a walk-through survey of the medical center. Each of these are described in detail below

### EHR data

We requested retrospective EHR data on all hospitalized patients at OSUWMC between January 2019 and April 2021. This included information on patient location and clinical history such as time of admission and discharge, time of transfer, demographics, and clinical conditions. The data were structured such that each row represented information for every unique room of each patient’s inpatient stay. Patients were classified as cases if they developed HO-CDI during their hospitalization, defined as a positive *C. diff* test after at least 3 days of an inpatient hospital stay.^14^ We excluded all hospitalizations for patients diagnosed with community-onset *C. diff* (CO-CDI) (defined as a positive C. diff test within the first 3 days of hospitalization) or those who stayed for less than 4 calendar days, as, by definition neither of these groups can be diagnosed with HO-CDI.

Matching was done using incidence density sampling without replacement,^15^ wherein a cohort member previously selected as a control was not eligible for further selection. For each case, the duration from time of admission (T_0_) to the time of sample collection (Te) was referred to as the **time of exposure** (Te-T_0_), and the date of sample collection as the **event date**. A patient was eligible to be a control if they did not have HO-CDI during their hospitalization or if they did, it was beyond the time of exposure for the matched case. Controls were matched to cases (4:1 ratio) who had the same month and year of inpatient admission and were *C. diff* free for the associated time of exposure.

**Figure 2.**
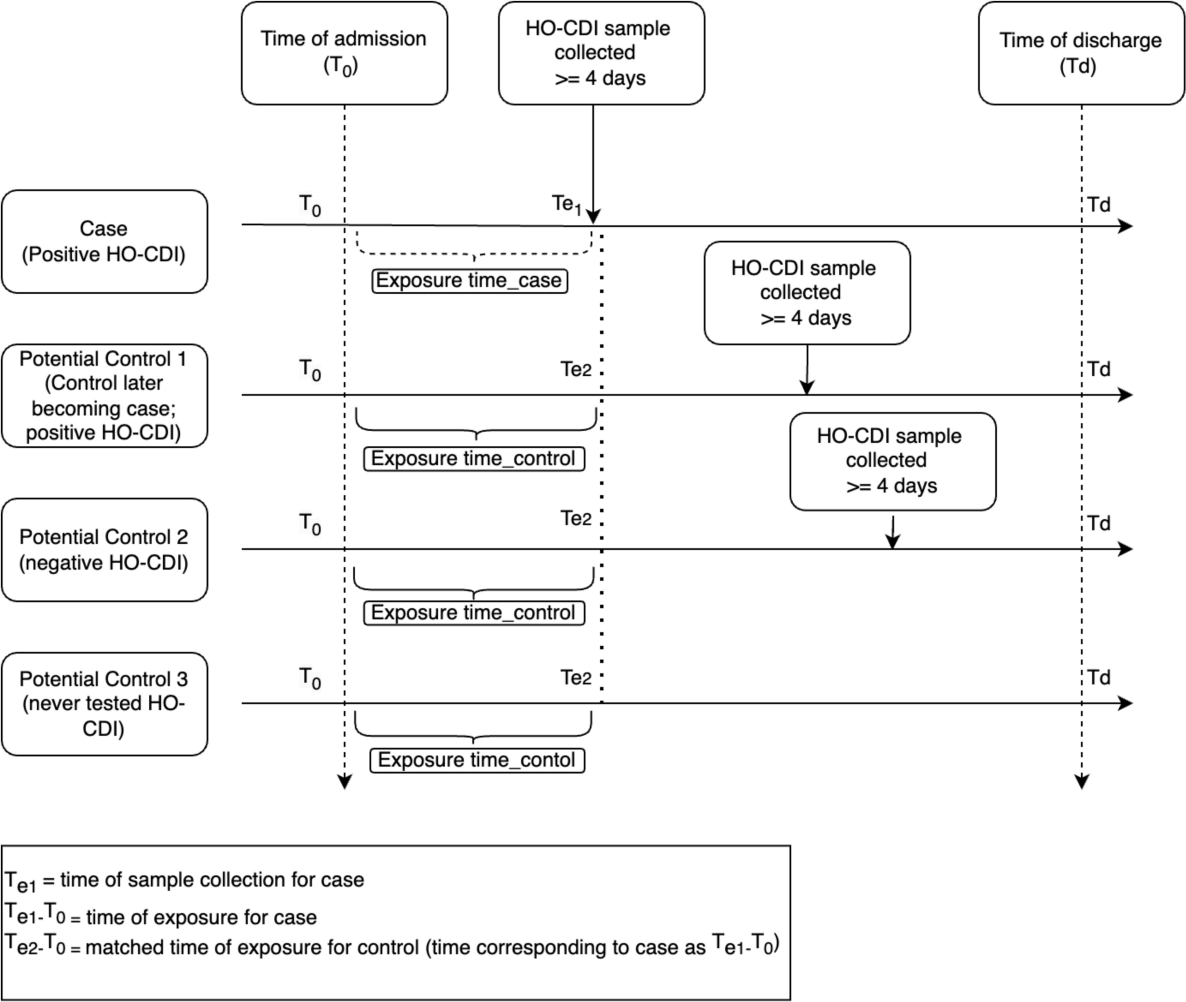
Types of potential controls for a case in the study.

Figure 2 illustrates the choice of possible controls.

For a case, any of the following individuals could serve as controls:

1) Control 1: A patient who did not have *C. diff* from admission through the exposure time of the case (Te_1_ -T_0_) (Te_1_ is the time of sample collection for the corresponding case), but later developed *C. diff*.

2) Control 2: A patient who was tested for *C. diff* during or after time of exposure (as associated with the case) but yielded a negative result and remained in the hospital for longer than Te_1_ -T_0._

3) Control 3: A patient who was never tested for *C. diff* and remained in the hospital for longer than Te_1_ -T_0._

The **cumulative exposure date** was set 24 hours prior to the event date. The **room of interest** for each case and control was the initial room the patient occupied on the cumulative exposure date. The features in the room of interest were used in the analyses to examine any associations with risk of infection.

### Survey data

For the second dataset, our team developed a walk-through survey using REDCap software to collect information on room features for all available inpatient rooms at OSUWMC. The medical center comprises several free-standing and adjoined buildings. The rooms included in this study were in 6 acute-care buildings in the medical center across two campuses. Further details about the survey can be found in previous work by our team.^2^ The survey was created with feedback from clinicians, Infection Preventionists (IPs), and those in Facilities Planning at OSUWMC. The survey data was stored in a REDCap database.^16,17^ At the time of the survey, there were 1404 inpatient beds at OSUWMC. We did not survey rooms in our psychiatric hospital, the unit devoted to incarcerated patients, labor and delivery, or the addiction medicine hospital. The finalized hospital-room survey was conducted in 1282 distinct rooms over several months of data acquisition. We merged survey and EHR data to get information on features in every room of the patient stay along with their clinical characteristics.

### Statistical analysis

Given the matching design, we conducted unadjusted conditional logistic regression to examine the association between each room feature and HO-CDI. In the final model, adjustments were made for patient factors, including high-risk antibiotic use in the past 30 days from event date (yes/no), clinical factors potentially associated with room assignment at our institution (eg, liver disease, organ transplant, inflammatory bowel disease, congestive heart failure, cancer), renal failure, previous diagnosis of *C. diff*, age at admission, and number of room transfers. The following antibiotics were classified as high risk: clindamycin, cephalosporins, carbapenems, fluoroquinolones, and piperacillin-tazobactam, while the remaining were classified as being low-risk: Sulfamethoxazole-Trimethoprim, aminoglycosides, tetracyclines, vancomycin, oxazolidinones, daptomycin, and other (antibiotics not included in previous classes). Oral vancomycin, fidaxomicin, metronidazole and topical antibiotics were not included in any of the classes. Antibiotics were categorized based on feedback from infectious disease subject-matter experts and findings from prior literature.^18–21^ Previous diagnosis of *C. diff* was defined as a positive *C. diff* result within a year prior to the admission date of the current hospitalization. The number of room transfers was defined as the total number of unique patient rooms that a patient was transferred during their hospitalization. This number did not include procedure rooms, only patient rooms. These variables were selected based on feedback from subject matter experts and previous literature.^21,22^ We conducted adjusted conditional logistic regression to explore the association between room features and HO-CDI, when controlling for patient and visit factors. All statistical analyses were conducted using Stata/SE version 17.0 software.^23^ The study was approved by the institutional review board at OSU.

## Results

### Sample characteristics

There was a total of 535 hospitalizations with HO-CDI in our dataset, of which 497 were retained for analysis after exclusions. These cases were matched to 1930 controls. In our entire cohort, the mean age was 61 years (SD = ±15.31), and 46% were female. The average time of exposure was 11 days, and 51 (2%) patients had CDI in the previous year. Table 1 provides characteristics of cases, matched controls, and the overall sample of the study.

**Table 1.** Characteristics of the entire sample, cases, and controls*

Variables	Cases (N = 497)	Controls (N = 1930)	Total (N = 2427)
**Age in years**, Mean ± SD	62 ± 14.52	60 ± 15.49	61 ± 15.31
**Sex**-Female, n (%)	246 (50%)	860 (45%)	1106 (46%)
**Race**-**Ethnicity,** n (%)			
1. Non-Hispanic White	392 (79%)	1391 (72%)	1783 (73%)
2. Non- Hispanic Black	74 (15%)	441 (23%)	515 (21%)
3. Hispanic	11 (2%)	40 (2%)	51 (2.10%)
4. Other	20 (4%)	58 (3%)	78 (3%)
**High-risk antibiotic use in past 30 d** - Yes, n (%)	419 (84%)	1416 (73%)	1835 (76%)
**Positive** ***C. diff*** **in the past year,** n (%)	21 (4 %)	30 (2%)	51 (2%)

* SD stands for standard deviation, n is the number of individuals, N = sample size

### Room characteristics

Of the 2427 patients in the study, there were 968 unique rooms of interest. Our study represented 968 of 1,404 (68.9%) of all inpatient hospital beds. As part of the survey, we investigated information on 58 room features, detailed in our previous work.^2^ Here we focus on 12 features that were of prior interest and contained sufficient variability within buildings to estimate associations. These features and their associated categories included : (1) the type of flooring in the patients’ room (linoleum, luxury vinyl tile, sheet vinyl tile, or vinyl composition), (2) type of door at the room entrance (double doors or doors with the ability to open both left and right sides for wider entrance, sliding door, dual swing door, or single swing doors), (3) presence of a cubical curtain in room (yes or no), (4) and its location (near the patient or near door), (5) type of paper towel dispenser (automatic or manual), (6) condition of the table in room (fair, good, like new), (7) type of marker board in room (digital or writable), (8) type of flooring in the attached bathroom (luxury vinyl tile, porcelain tile, epoxy or vinyl composition tile), (9) type of shower unit (without separate shower space in the bathroom (pan units) or with separate shower space), (10) type of toilet unit (floor mount or wall mount), (11) type of bathroom door (normal swing door or sliding door), and (12) handle type for bathroom door (lever, sliding door handle, or other).

### Association with HO-CDI

Unadjusted analyses indicated significant associations with HO-CDI for rooms with the following features: sheet vinyl room flooring (marginal), cubical curtain near patient, manual paper towel dispenser, separate shower units, wall mounted toilets, bathrooms with sliding doors, and bathrooms with sliding door handles.

Results from adjusted analyses suggested protective effects for rooms with cubical curtains near the patient (OR = 0.705, 95% CI = 0.549-0.906) compared to those near the door, rooms with separate shower units (OR = 0.674, 95% CI = 0.528–0.861) compared to those with a shower pan unit, and rooms with wall mounted toilets (OR = 0.749, 94% CI = 0.592–0.950) compared to those with floor mounted toilets. Rooms with manual paper towel dispensers had increased odds of HO-CDI (OR = 1.334, 95% CI = 1.053–1.691) compared to those with automatic towel dispensers.

Bathrooms with sliding doors and sliding door handles were also associated with reduced risk of infection compared to swing doors and lever knobs. Table 2 provides the list of room features and their results as observed in the study.

**Table 2.** Odds ratio for unadjusted and adjusted models across the selected room features

	Unadjusted		Adjusted	
Variable	Odds ratio (95% CI)	p value	Odds ratio (95% CI)	p value
**Type of floor in main room** (ref-Linoleum)				
1. Luxury vinyl tile	0.748 (0.491–1.140)	0.177	0.750 (0.483–1.165)	0.201
2. Sheet vinyl	0.723 (0.519–1.008)	0.056	0.705 (0.498–1.000)	0.050*
3. Vinyl composition	0.886 (0.709–1.114)	0.294	0.899 (0.703–1.149)	0.395
**Type of door** (ref-Double/Wide function door)				
1. Sliding door	0.961 (0.683–1.350)	0.817	0.844 (0.589–1.210)	0.357
2. Swing door/dual	0.776 (0.452–1.334)	0.359	0.771 (0.444–1.340)	0.357
3. Swing door/single	1.104 (0.836–1.460)	0.483	1.081 (0.807–1.449)	0.602
**Cubical curtain in room** (ref-no)				
1.Yes	0.849 (0.646–1.115)	0.239	0.802 (0.607–1.062)	0.124
**Location of cubical curtain** (ref-near door)				
1. Near patient	0.732 (0.574-0.936)	0.013**	0.705 (0.549-0.906)	0.006**
**Type of paper towel dispenser** (ref-automatic)				
1. Manual	1.273 (1.022–1.584)	0.031**	1.334 (1.053–1.691)	0.017**
**Table condition** (ref-fair)				
1. Good	0.961 (0.746–1.237)	0.756	0.987 (0.758–1.288)	0.928
2. Like new	0.785 (0.545–1.131)	0.195	0.762 (0.523–1.112)	0.160
**Type of marker board** (ref-digital)				
Writable	1.063 (0.739–1.531)	0.742	1.158 (0.795–1.688)	0.444
**Floor type in bathroom** (ref-luxury vinyl tile)				
1. Porcelain tile	1.060 (0.544–2.070)	0.170	1.044 (0.525–2.076)	0.902
2. Epoxy	0.745 (0.385–1.445)	0.385	0.701 (0.358–1.372)	0.300
3. Vinyl composition tile	0.477 (0.123–1.852)	0.285	0.469 (0.1191.851)	0.280
**Type of shower** (ref-pan shower unit)				
1. Separate shower unit	0.691 (0.548–0.872)	0.002**	0.674 (0.528–0.861)	0.002**
**Type of toilet** (ref-floor mounted)				
1. Wall mounted	0.748 (0.598–0.937)	0.012**	0.749 (0.592–0.950)	0.017**
**Door type for bathroom** (ref-normal swing door)				
1. Sliding door	0.681 (0.500–0.927)	0.014**	0.593 (0.432–0.816)	0.001***
**Handle type for bathroom door** (ref-lever knob)				
1. Sliding door handle	0.677 (0.498–0.922)	0.013**	0.593 (0.431–0.815)	0.001***
2. Push handle or turn knob	0.788 (0.172–3.601)	0.759	0.918 (0.198–4.255)	0.913

ref = reference category; CI = confidence interval; *p < 0.10, **p < 0.05, ***p < 0.001

## Discussion

Our results suggest significant associations between specific room features and risk of HO-CDI, even after adjusting for patient and visit factors. Previously, we presented results from an exploratory study that examined the relationship between hospital room features and HO-CDI in a smaller sample.^2^ In this study, we extended our investigation to examine the risk of HO-CDI when accounting for potential confounders, using a nested case–control study design, and on a greater proportion of inpatient rooms at OSUWMC. We also accounted for temporal variation by matching cases and controls on the same admission month and year, along with incidence density sampling to account for length of stay. A strength of this study included the rigorous study design and the inclusion of factors associated with room assignment in the model. The study also made use of a unique data source, a detailed walk-through survey of an in-patient hospital.

Our findings warrant further investigation to determine how they might be related to HO-CDI risk. We saw marginal association between flooring type and risk of *HO-CDI.* Rooms with sheet vinyl flooring had lower odds of infection compared to linoleum, in alignment with previous literature. Previous studies indicated that sheet vinyl floorings have sterile properties^24,25^ which might contribute to the protective effect. Moreover, linoleum has seams and is more impervious to water than vinyl, thus potentially trapping moisture and harboring organisms.^26^ Cubical curtains often harbor infectious organisms, however there is no literature that examines the impact of the location on the risk of infection.^27^ It is possible that curtains further away from patients may be more likely to come in contact with healthcare workers and visitors walking in and out of rooms and potentially contaminating them. To our knowledge, ours is the first study to examine this relationship and further exploration of this feature is warranted.

We found that rooms with touchless or automatic paper towel dispensers had a protective effect on risk of HO-CDI compared to those with manual dispensers. Automated paper towel dispensers remove a high touch point, and thus the likelihood of transmission of organisms from individuals to surfaces and from surfaces to other individuals.^28^ Other room features associated with risk of infection were related to bathrooms and toilets. A separate shower unit was protective when compared to a shower pan unit. This could reflect an easier route of contamination throughout the bathroom when the shower and toilet areas are not separated. Literature has reported a higher concentration of microbes on toilet floors compared to the back of toilets and toilet walls.^29^ Moreover, presence of a toilet base on the floor compared to those mounted on the walls could provide additional surface area for microbes to reside and thus increase the chances of transmission from the environment. Also, presence of a toilet mount may make it difficult to clean the underlying flooring, thus contributing to the increased risk.

There are several limitations to this study. First, it was conducted at a single tertiary care hospital, which raises concerns about generalizability. Despite collecting many variables on each room, we had to limit our list of room features because of the lack of intra-building variability at our institution. It is challenging to assess the impact of a single room feature in a complex hospital environment. Our analysis showed an association between some room features and HO-CDI but that does not mean that that room feature is the cause of the increased risk. The analysis was based on EHR data and is limited by what is recorded, so unmeasured confounding is possible. In addition, patients are rarely limited to their rooms while hospitalized and can interact with the built environment in multiple areas of the hospital, which was not accounted for in this study design. Despite adjusting for the number of transfers, we were not able to account for all room and building features that patients came into contact during their stay. Therefore, we restricted our analysis to the room where sample was collected or the one likely to be associated with disease contraction. Another factor that could impact the outcomes of the study would be variations in cleaning protocols between rooms or buildings. However, at our institution, these protocols do not differ and as a result were not considered in the analysis. Lastly, our data collection occurred during the COVID-19 pandemic, which led to widespread changes in clinical practices. However, matching cases and controls on the month and year of admission minimizes this effect.

## Conclusion

Results suggest possible association between specific room features and HO-CDI, which could be further investigated with techniques like environmental sampling. The outcomes of this study not only pinpoint specific high-risk surfaces but also offer valuable insights for targeted intervention measures. Additionally, these results could inform room allocation practices and guide the implementation of targeted cleaning protocols to effectively disinfect high-risk surfaces. Future research could explore the impact of novel disinfection methods, disinfection of shared equipment, hand hygiene adherence, antimicrobial coatings, varying disinfecting agents, and behavioral interventions.
